# Childbearing and Economic Work: The Health Balance of Women in Accra, Ghana

**DOI:** 10.1007/s10995-015-1839-2

**Published:** 2015-11-04

**Authors:** Philippa Waterhouse, Allan G. Hill, Andrew Hinde

**Affiliations:** Faculty of Health and Social Care, Open University, Milton Keynes, MK6 7AA UK; Department of Social Statistics and Demography, University of Southampton, Southampton, UK; Southampton Statistical Sciences Research Institute, University of Southampton, Southampton, UK

**Keywords:** Ghana, Health, Well-being, Women’s roles, Work and family

## Abstract

*Objectives* This study aims to investigate (1) whether the health of working women with young children differs from that of working women without young children, and (2) which social factors mediate the relationship between economic and maternal role performance and health among mothers with young children. *Methods* The analyses uses panel data from 697 women present in both waves of the Women’s Health Study for Accra (WHSA-I and WHSA-II); a community based study of women aged 18 years and older in the Accra Metropolitan Area of Ghana conducted in 2003 and 2008–2009. Change in physical and mental health between the survey waves is compared between women with a biological child alive at WHSA-II and born since WHSA-I and women without a living biological child at WHSA-II born in the interval. To account for attrition between the two survey waves selection models were used with unconditional change score models being used as the outcome model. *Results* We found in our sample of working women that those who had a child born between WHSA-I and WHSA-II who was still alive at WHSA-II did not experience a change in mental or physical health different from other women. Among working women with young children, educational status, relationship to the household head and household demography were associated with change in mental health at the 5 % level, whilst migration status and household demography was associated with change in physical health scores. *Conclusion* The results suggest there are no health penalties of combining work and childbearing among women with young children in Accra, Ghana.

## Significance

*What is Already Known on This Subject?* Research in the West has found contradictory results concerning the impact of women’s combination of motherhood and economic activity on their own health with positive, negative and insignificant results being found. A limitation of this research is its narrow geographical focus having been conducted mainly in Anglo-Saxon countries.

*What This Study Adds?* This paper contributes through exploring the influence of maternal multiple roles in the sub-Saharan African context. We found there were no health penalties of combining economic work with having young children among women in Accra, Ghana.

## Introduction

Since the 1960s there has been a substantial volume of literature investigating the interface between work and family [15], with early writing focusing particularly on the consequences of maternal employment for both women and other family members [34]. A limitation of this research is its narrow geographical focus, having been conducted mainly in Anglo-Saxon countries [3]. Nonetheless, the experience of combining work and children is likely to vary with differences in employment and family structures, cultural values and national policies [8]. Focusing on sub-Saharan Africa (SSA), there has been concern about women’s intensifying workloads resulting from the impact of structural readjustment on economic activity [33, 38], and from the consequence of changing patterns of family support on women’s domestic responsibilities [30–32].

Whilst research has investigated the association between maternal employment and child health in this context [1, 41], relatively little literature exists on the implications of maternal employment for maternal health. Using panel data this study aims, first, to investigate the health impact of motherhood on working women in the Accra Metropolitan Area (AMA) of Ghana; and, second, to examine the associations between socio-economic characteristics and household structures and change in health among working mothers with young children. Female engagement in economic activity has been strong historically in Ghana [33], and recent figures show female labor force participation to be relatively high (67 %) [47]. The return of women to work soon after giving birth in the AMA [22] suggests that this population is combining economic activity with having young children. We focus on mothers of young children as parental demands have been highlighted as an important antecedent of work-family role conflict [21, 43].

### Previous Research and Study Hypothesis

Research on the implications of multiple roles for individual health has been stimulated by the role strain and scarcity hypotheses [19] which posit that the engagement in multiple roles is detrimental to health as increases in demands result in the exhaustion of resources. In the absence of overload, conflict can have negative implications for health when the performance of one role makes the negotiation of a second role difficult [20]. Yet, taking a role enhancement, accumulation or expansion approach [37], engagement in multiple roles is seen as beneficial for health. For example, maternal engagement in economic activity may result in increases in living standards and nutrition of women through income earned. Women’s ability to balance both their roles may result in emotions such as satisfaction, self-esteem, happiness and positive identity. Lastly, women’s involvement in mothering may provide compensation where dissatisfaction is experienced in work, or vice versa.

The quantitative literature presents contradictory results with negative [6, 13, 23], positive [11] and non-significant impacts [5, 28, 42] of combining motherhood and economic activity on health being reported. In the context of Ghana studies have linked women’s combination of their economic and maternal roles to negative health outcomes. Avotri and Walters [9] found women frequently attributed their ailments to their work and family demands. Sackey and Sanda [36] found the maternal role to be statistically associated with mental health as measured by symptoms of depression, anxiety and stress. However, their study was limited to professional and managerial women, a group that does not represent the majority of working women in Ghana.

From the literature on maternal employment and health in Ghana, and the wider literature on changes in women’s roles in SSA discussed in the introduction we can derive our first hypothesis:

#### **H1**

Having a young child results in negative implications for the health of working women.

Elliot and Huppert’s [16] study of the effect of employment on the health of British mothers found differences based on whether mental or physical health was considered. Consequently, this study investigates the impact of motherhood on both these dimensions of health.

Second, we aim to examine whether socio-economic characteristics and household structure mediate the relationship between multiple role performance and health among women with young children. Arber [5] emphasises the importance of the social, material and structural conditions under which roles are performed. Hence our second hypothesis is:

#### **H2**

Socio-economic characteristics and household structure is associated with health among working mothers of young children.

Our analysis deepens understanding of the linkages between multiple roles and health through allowing for the identification of possible health variations between women performing multiple roles [24]. Socio-economic characteristics considered by this study are change in wealth, partnership status, religion, educational attainment, ethnicity, and whether a woman was born in the Greater Accra region.

In studies of Western populations the partnership status of women is given considerable attention with lone mothers in employment, especially full time, frequently found to have worse self-reported health than employed mothers of other marital statuses [24, 28]. However, caution must be exercised before applying these ideas to different societies where the prevalence, meaning and responsibilities of marriage may differ [24]. In the Ghanaian context, for example, ethnicity is closely associated with lineage systems and can determine relationships between fathers and children [2], and between women and prenatal kin and husbands after marriage [39].

Migration status has also been linked to social support [25], therefore those who are migrants to the AMA may experience more restricted availability of support to help deal with the demands of combining young children with economic activity. The Western literature focuses on social class membership [5, 13, 16, 28] which has been seen as an indicator of a household ability to purchase time-saving services, absence of financial worry, nature of employment and consequent job satisfaction. We include educational attainment which could be seen as an indicator of social class. A decline in household wealth could be a source of strain to mothers through financial worry, but could also be associated with the use of resources by the mothers to purchase services to cope with work and family. We include religion which could mediate the effect of motherhood on health as a form of coping [29], for example through regulating emotions or providing access to instrumental support networks.

In addition to socio-economic characteristics we examine the possible mediating influence of a woman’s relationship to the household head and household demography in terms of number of individuals of each age and sex. Annim et al.’s [4] analysis of household compositions of children under five in Ghana reveals that a diversity of arrangements exists ranging from extended family structures to lone mothers. The number of young children in the household may mean additional demands placed on a woman, whilst the presence of older female children and women may suggest additional sources of assistance. Relationship to household head can affect access and control of household resources [17], and consequently women’s ability to use these to cope with their demands of combining motherhood with economic activity.

## Methods

### Data

We use data from the Women’s Health Study for Accra (WHSA); a community-based panel study of women aged 18 years and older (at wave I) resident in the AMA. Wave I of data collection (WHSA-I) was conducted in 2003 and Wave II (WHSA-II) in 2008–2009. The project was led by the Institute of Statistical, Social and Economic Research (ISSER), University of Ghana, in collaboration with the Harvard School of Public Health.

### Sample

The relationship between multiple roles and health is potentially endogenous with engagement in roles being dependent on health status, and health being affected by role performance [5]. As the participation of healthy individuals in the labour force is widely documented by the literature [6, 26, 44] we restrict our sample to women working at both waves. Due to the focus on births the sample was restricted to women of reproductive age (18–49 years) at the time of WHSA-I (n = 697). To investigate the health impact of motherhood on working mothers, a birth variable (BIRTH) was created using information from the birth histories. This variable equaled 1 for women who had a child alive at WHSA-II who was born since WHSA-I (n = 207), and 0 for all other women (n = 490). The engagement of women in the parenthood role may also involve selection bias; however we found no significant difference when comparing the mental and physical summary baseline scores of our two groups of women. Only 1.9 % of the sample experienced the death of a child born in the survey interval and some of these had another child born in the survey interval who was still alive at WHSA-II. Regression analysis revealed that child mortality was not significantly associated with change in maternal physical or mental health.

### Variables

#### Dependent Variables

The SF-36, one of the most extensively used and tested instruments for measuring health internationally, was used to construct the outcome measures of health. The SF-36 consists of 36 questions that can be used to form eight scales of health (physical functioning, role limitations due to physical problems, bodily pain, general health, vitality, social functioning, role limitations due to emotional problems and emotional wellbeing) [45]. The health scales can also be used to create two summary measures, the mental component summary score (MCS) and the physical component summary score (PCS), according to the physical and mental variance that the concepts have in common. The RAND-36 methodology was used to construct the eight health domains and principal component analysis to construct the summary measures (RAND Cooperation [35]. As the research was interested in health over time a change score was constructed by subtracting WHSA-I component scores from WHSA-II component scores.

#### Socio-economic and Household Demography Covariates

Based on a comparison of wealth quintile membership at WHSA-I and WHSA-II a three category variable was created based on whether wealth status in WHSA-II was lower, higher or the same as in WHSA-I. Six categories of partnership dynamics were considered: (1) never-married at both waves, (2) married at both waves, (3) separated, divorced or widowed at both waves, (4) married or never-married in WHSA-I, separated, divorced or widowed in WHSA-II, (5) never-married in WHSA-I, married in WHSA-II, and (6) separated, divorced or widowed at WHSA-I, married at WHSA-II. It should be noted that in the survey interval women may have experienced multiple transitions, for example a woman married at both time points was not necessarily married to the same man. Religious beliefs and educational attainment showed little difference at the two survey waves and consequently their values as recorded in WHSA-I were included in the analysis. Religion was categorized as: (1) Christian, (2) Muslim, (3) other (those with no religion and traditionalists). Educational attainment was grouped as: (1) none, (2) primary, (3) Junior Secondary School (JSS), and (4) Senior Secondary School (SSS) and higher. Ethnicity is a time invariant covariate with the three major ethnic groups in Accra being distinguished. Whether a woman was born in the Greater Accra region was also considered. Using the household roster variables representing household structure at the time of the WHSA-I were created. The sample distribution according to socio-economic characteristics and household demography is shown in Table 1.

**Table 1:** Distribution of sample in terms of socio-demographic and economic characteristics (n = 697)

Covariate change	% of sample		
	Total	Birth	No birth
Wealth			
Wealth status lower in WHSA II than WHSA I	38.16	36.23	38.98
Wealth status higher in WHSA II than WHSA I	24.25	25.60	23.67
Wealth status same	37.59	38.16	37.35
Partnership dynamics			
Never married WHSA I and WHSA II**	21.23	6.28	27.55
Married WHSA I and WHSA II**	40.03	50.72	35.51
Separated, divorced or widowed WHSA-I and WHSA II	10.04	2.90	13.06
Never married in WHSA I, married in WHSA II**	11.62	23.67	6.53
Sep, div or wid in WHSA I, married in WHSA II	10.63	9.18	11.22
Never married/married in WHSA I, sep, div or wid in WHSA II	6.46	7.25	6.12
Baseline characteristics			
Religion			
Christian	84.36	85.71	81.16
Muslim	11.76	10.41	14.98
Other	3.87	3.88	3.86
Education			
None	9.61	9.39	10.14
Primary	11.05	10.00	13.53
Junior Secondary School	50.65	49.18	54.11
Senior Secondary School or higher	28.69	31.43	22.22
Ethnicity			
Akan	37.07	36.73	37.86
Ga	37.50	38.16	35.92
Ewe	12.07	12.24	11.65
Other	13.36	12.86	14.56
Relationship to the household head**			
Head	18.79	21.84	11.59
Wife	34.86	32.04	41.55
Daughter/daughter in-law	27.69	11.02	29.95
Other	11.19	11.02	11.59
Not specified	7.46	8.37	5.31
Total	100 (n = 697)	100 (n = 207)	100 (n = 490)
Age (years)**	30.38	31.71	27.21
Mental component score	52.35	52.28	52.52
Physical component score	52.51	53.53	52.47
Household composition			
Children 0–5 years**	0.48	0.40	0.68
Children 6–11 years	0.56	0.58	0.52
Females 12–15 years	0.22	0.24	0.19
Males 12–15 years	0.19	0.22	0.15
Females 16–54 years	2.02	2.04	1.99
Males 16–54 years	0.86	0.83	0.92
Females 55 years+	0.18	0.16	0.22
Males 55 years+	0.15	0.15	0.15

** Statistically significant difference between women who had a child alive at the time of the WHSA II born in the survey interval (birth) and women who did not have a child alive at the time of the WHSA II born in the survey interval (no birth) 2. Where individuals stated they were of a separated, widowed and divorced status in the WHSA-I and a never married status in the WHSA-II, it was assumed this latter status was married. This applied to 1.1 % of the sample. Where individuals stated they were married in the WHSA-I and never married in the WHSA-II, this latter status was assumed to be separated, divorced or widowed. This applied to 1.3 % of the sample. 3. It should also be noted that the partnership dynamic variables represent status as known at the WHSA-I and WHSA-II and do not capture multiple transitions, for example women married at the time of both surveys may not necessarily be married to the same man

## Analysis

To test hypothesis 1, the change in health between the survey waves of women who had a child born between WHSA-I and WHSA-II and still alive at WHSA-II (n = 207) was compared to the change in health of other women (n = 490). To test hypothesis 2, the analysis was restricted to women with a child born in the survey interval (n = 207) and the socio-economic characteristics and household structure variables were considered as independent variables. To take account of attrition between the survey waves a two-step procedure was used involving the specification of two models; the selection model and the outcome model. The selection model investigates the mechanism of selection of women into both waves of the survey. Selection is determined by a linear combination of observed and unobserved covariates [10]. Through the specification of this selection function as a probit model, parameters are used to estimate a measure of selection bias for every case (the inverse Mills ratio (IMR). The unconditional change score model, the outcome model, was estimated for cases that selected into the both waves of the data. The IMR was incorporated into this model as a covariate. In order to account for the two-step method bootstrapping was used to correct standard errors.

There is little guidance available concerning the covariates that should be used to estimate the IMR, consequently several variations of the probit model were tested to investigate the sensitivity of the outcome model to different selection function specifications. The first selection model (SM1) incorporated socio-economic and demographic characteristics of individuals as recorded at the WHSA-I. In selection model 2 (SM2) two instrumental variables gained from the WHSA-I’s ‘paradata’ were used; participation in the medical examination and interview co-operation. These are assumed to be strong predictors of selection. Participation in the medical examinations required women to attend the outpatient clinic at the Korle Bu Teaching Hospital. It involved fasting prior to the appointment and was noted by the research team to be a demanding process with women kept waiting. On the one hand this experience could have led to individuals being reluctant to take part in another wave of the survey, whilst conversely since women had invested time in the survey it could have meant they felt bound to continue and not to lapse between waves. Measures of interview compliance and cooperation have been found to be good predictors of drop-out in other surveys [40]. A third instrumental variable, how long individuals had been resident in their WHSA-I household, was also included. Individuals resident in their households for shorter periods of time may be more mobile and therefore may have been harder to locate for the WHSA-II. Post-estimation Wald tests show these instrumental variables do not show significance in either the PCS or MCS outcome models; however they do show a significant relationship with selection into both waves of the survey. Selection model 3 (SM3) took a mechanical approach incorporating only significant predictors in the estimation of the IMR. Results from the selection models can be found in Table 2. There is no one criterion to select the ‘best choice’ specification, however the three models are nested so their fit to the data can be compared using the log likelihood ratio test. According to this test SM2 fits the data best.

**Table 2:** Women’s socio-economic and household demographic predictors of selection into both waves of the WHSA among women of reproductive age present in the WHSA I (n = 1285)

	SM1	SM2	SM3
Log likelihood	−1249.71	−1188.40	−1416.05
Variables			
Age	0.0121*	0.0015	–
Wealth			
Poorest	0.9894**	−0.5496**	−0.5261**
Fourth	−0.1699	−0.1544	−0.2002*
Middle^a^			
Second	−0.2194*	−0.2465**	−0.2108*
Richest	−0.7331	−0.0996	−0.0894
Marital status			
Married^a^			
Never married	−0.1187	−0.1168	–
Separated/divorced/widowed	−0.0430	−0.0313	–
Religion			
Christian^a^			
Muslim	0.1552	0.1203	–
Other	−0.0563	−0.1143	–
Ethnicity			
Ga^a^			
Akan	−0.4126**	−0.3068**	−0.3061**
Ewe	−0.2987**	−0.2083*	−0.0262**
Other	−0.3241*	−0.2091	−0.1557
Education			
None	−0.2685**	−0.2813**	−0.1925*
Primary	−0.1240	−0.1047	−0.0795
Junior Secondary School^a^			
Senior Secondary School or higher	−0.0012	0.0168	0.0238
Relation to head of household			
Head	−0.0836	−0.0716	–
Wife^a^			
Daughter/daughter in-law	0.1077	0.0263	–
Other	−0.2432*	−0.2231	–
Not specified	−0.0744	−0.1201	–
Household Demographics			
Number of children 0–5 years			
0^a^			
1	0.0797	0.0665	–
2+	0.0608	0.0560	–
Number of children 6–11 years			
0^a^			
1	0.0766	0.0650	0.0561
2+	0.2865**	0.2916**	0.2963**
Number of females 12–15 years			
0^a^			
1+	0.0590	0.0305	–
Number of males 12–15 years			
0^a^			
1+	0.0991	0.0976	–
Number of females 16–54 years			
1^a^			
2	0.0716	0.0515	–
3	0.1494	0.0868	–
4+	0.3507**	0.3054**	–
Number of males 16–54 years			
0	0.0935	0.0803	–
1^a^			
2	0.1772	0.1236	–
Number of females 55 years +			
0^a^			
1+	0.2142*	0.1554	–
Number of males 55 years +			
0^a^			
1+	−0.1398	−0.1277	–
Wellbeing			
PCS	−0.0081*	−0.0054	–
MCS	0.0096**	0.0086*	–
Participation in medical examination			
Yes^a^			
No	–	0.3910**	0.4378**
Cooperation			
Excellent	–	−0.1860**	−0.2271**
Good^a^			
Poor	–	−0.1237	−0.2054
Years resident at household			
0–4 years^a^			
5–9 years	–	0.2267*	0.2219**
10–19 years	–	0.3838**	0.4540**
20–29 years	–	0.5295**	0.5450**
30+ years	–	0.5396**	0.6089**

*PCS* physical component summary score, *MCS* mental component summary score, *SM1* selection model 1, *SM2* selection model 2, SM3 selection model 3

* Significance at the 5 % level and ** 1 % level

^a^Reference category, all variables refer to their values at baseline (WHSA-I)

## Results

Hypothesis 1 supposes that having young children will have negative implications for the mental and physical health of working women. To test this hypothesis, six change-score models were fitted to the full sample (n = 697); three using change in mental health as an outcome and incorporating a different IMR to control for attrition, and three using change in physical health as the outcome. The variable BIRTH (had a child between WHSA-I and WHSA-II who was still alive at WHSA-II) was included to compare the change in health between our two groups of women. Our results found BIRTH to be insignificant regardless of the dimension of health considered and the selection specification used (Table 3). Therefore hypothesis 1 is not supported as in our sample of working women those that had a child born in the survey interval did not experience a change in their mental or physical health significantly different to those without a child born in the survey interval.

**Table 3:** Parameter estimates (point difference) of change in mental and physical component summary scores between the WHSA I and WHSA II between women who had a child alive at WHSA II born in the survey interval and women who did not have a child alive in the WHSA II, controlling for attrition between survey waves (n = 697)

	Selection specification								
	SM1			SM2			SM3		
	β	95 % CI		β	95 % CI		β	95 % CI	
		Lower	Upper		Lower	Upper		Lower	Upper
Change in MCS									
Birth	−0.46	−2.97	2.04	−0.69	−3.24	1.86	−0.60	−3.16	1.96
Change in PCS									
Birth	0.37	−1.54	2.29	0.47	−1.47	2.42	0.49	−1.46	2.42

*MCS* mental component summary score, *PCS* physical component summary score Analysis includes socio-economic and household demography variables as controls

It may be, however, that the birth of a child influences health through causing a change in wealth, for example, scaling back working hours or changing occupations are possible work-family management strategies that could affect the household economy. Wealth could also be affected by the cost of a new child. Bivariate analysis between change in health and change in wealth, whilst controlling for attrition, revealed that change in wealth was associated with change in mental health, but not physical health. However, Chi square analysis showed that change in wealth was not significantly associated with our BIRTH variable suggesting that a birth triggers no wealth-induced shock to the household.

Hypothesis 2 suggests that, among working women with young children, socio-economic characteristics and household demography will mediate the relationship between maternal and work role performance and health. To examine this hypothesis the sample was restricted to women who had a child alive at WHSA-II who had been born in the survey interval (n = 207). Six models were fitted with mental and physical health with the outcomes, each with a different IMR included to control for attrition. The results (Tables 4 and 5) lend support to hypothesis 2 with particular socio-economic categories and household compositions showing significant associations with change in health.

**Table 4:** Parameter estimates (point difference) of change in mental health among women who had a child alive born in the survey interval according to socio-economic characteristics and household demography, controlling for attrition between survey waves (n = 207)

	SM1			SM2			SM3		
	β	95 % CI		β	95% CI		β	95 % CI	
		Lower	Upper		Lower	Upper		Lower	Upper
Wealth									
Wealth status lower in WHSA-II than WHSA-I^a^									
Wealth status higher in WHSA-II than WHSA-I	1.91	−3.54	7.35	2.70	−2.66	8.06	2.88	−2.50	8.27
Wealth status consistent	2.60	−2.26	7.47	2.46	−2.49	7.41	3.03	−1.90	7.97
Partnership dynamics									
Never married WHSA-I and WHSA-II	6.37	−3.09	15.83	7.37	−2.17	16.91	7.35	−2.29	17.00
Married^a^ WHSA-I and WHSA-II									
Separated, divorced or widowed WHSA-I and WHSA-II	1.08	−11.35	13.52	0.99	−11.55	13.54	1.68	−10.91	14.26
Never married in WHSA-I, married in WHSA-II	3.85	−3.06	10.77	4.69	−2.45	11.84	5.36	−1.74	12.47
Sep, div or wid in WHSA-II, married in WHSA-II	0.65	−7.03	8.32	0.22	−7.58	8.02	0.89	−6.91	8.70
Never married/married in WHSA-I, sep, div or wid in WHSA-II	1.52	−6.67	9.71	−0.23	−9.16	8.71	0.48	−8.47	9.44
Change in residence WHSA-I and WHSA-II									
Yes	−1.66	−6.45	3.13	−1.76	−6.68	3.15	−1.58	−6.52	3.35
No^a^									
Religion									
Christian^a^									
Muslim	6.69	−2.89	16.28	6.81	−2.89	16.52	5.90	−3.78	15.57
Other	−2.14	−12.56	8.28	−1.88	−12.40	8.65	−2.08	−12.68	8.52
Highest educational attainment WHSA-I									
None	−9.26**	−16.66	−1.85	−8.98**	−16.37	−1.59	−7.74**	−15.11	−0.38
Primary	−0.39	−7.15	6.36	0.37	−6.33	7.07	1.79	−4.82	8.39
JSS^a^									
SSS +	−0.61	−5.69	4.47	−1.47	−6.77	3.83	−1.48	−6.81	3.84
Ethnicity									
Ga^a^									
Akan	−1.49	−8.85	5.87	0.44	−5.69	6.57	2.07	−3.96	8.10
Ewe	0.70	−7.15	8.54	1.33	−6.29	8.96	3.11	−4.58	10.80
Other	−7.05	−17.37	3.27	−6.22	−16.34	3.90	−4.91	−14.98	5.16
Born in the Greater Accra region									
Yes	4.01	−0.90	8.91	4.90*	−0.23	10.03	4.79*	−0.37	9.94
No^a^									
Age at WHSA-I	0.14	−0.36	0.64	0.17	−0.33	0.67	0.23	−0.44	0.56
Relationship to household head									
Head	4.17	−3.99	12.33	4.12	−3.90	12.14	5.23	−2.69	13.14
Wife^a^									
Daughter/daughter in-law	−11.40***	−19.85	−2.95	−12.74***	−21.50	−3.98	−12.92***	−21.71	−4.12
Other	−9.73*	−19.77	0.32	−9.46*	−19.12	0.19	−7.77	−17.19	1.65
Not specified	−11.58**	−22.07	−1.10	−11.80*	−22.44**	−1.17	−11.78**	−22.48	−1.08
Household demographics									
Number of children aged 0–5 years									
0^a^									
1	2.80	−2.12	7.72	2.71	−2.34	7.75	2.89	−2.21	7.99
2+	−1.50	−8.42	5.43	−1.30	−8.32	5.73	−1.17	−8.24	5.90
Number of children aged 6–11 years									
0^a^									
1	−0.08	−5.36	5.20	−0.02	−5.46	5.42	0.16	−5.30	5.63
2+	−4.34	−11.69	3.01	−4.92	−12.07	2.22	−6.67*	−13.75	0.41
Number of females aged 12–15 years									
0^a^									
1+	0.11	−5.77	5.99	−0.63	−6.61	5.35	−0.36	−6.40	5.68
Number of males aged 12–15 years									
0^a^									
1+	−1.88	−8.52	4.75	−1.93	−8.74	4.88	−1.52	−8.44	5.39
Number of females aged 16–54 years									
1^a^									
2	5.29	−1.23	11.81	6.13*	−0.77	13.02	5.17	−1.74	12.08
3	9.82**	2.19	17.44	10.44***	2.74	18.14	9.08***	1.42	16.73
4+	4.18	−4.55	12.92	3.79	−4.54	12.11	1.45	−6.28	9.18
Number of males aged 16–54 years									
0	2.07	−3.30	7.44	2.68	−2.86	8.22	2.28	−3.26	7.81
1^a^									
2+	10.05**	2.75	17.34	10.49***	2.76	18.21	9.39**	1.73	17.05
Number of females aged 55 years+									
0^a^									
1+	3.81	−2.33	9.94	3.71	−2.35	9.76	3.26	−2.78	9.31
Number of males aged 55 years+									
0^a^									
1+	3.19	−3.08	9.47	2.51	−4.02	9.04	2.69	−3.88	9.27
Constant	−17.83	−39.92	4.25	−17.77	−37.59	2.04	−9.96	−29.52	9.60
IMR	11.43	−8.38	31.24	7.83	−4.45	20.11	−0.64	−11.96	10.69

*JSS* Junior Secondary Schooling, *SSS* Senior Secondary Schooling, *SM1* inverse Mills ratio calculated using selection model 1, *SM2* inverse Mills ratio calculated using selection model 2, *SM3* inverse Mills ratio calculated using selection model 3

*** Significance at the 1 % level, ** 5 % level, * 10 % level

^a^Reference category

**Table 5:** Parameter estimates (point difference) of change in physical health among women who had a child alive born in the survey interval according to socio-economic characteristics and household demography, controlling for attrition between survey waves (n = 207)

	SM1			SM2			SM3		
	β	95 % CI		β	95 % CI		β	95 % CI	
		Lower	Upper		Lower	Upper		Lower	Upper
Wealth									
Wealth status lower in WHSA-II than WHSA-I^a^									
Wealth status higher in WHSA-II than WHSA-I	2.88	−2.50	8.27	2.62	−1.49	6.73	2.59	−1.50	6.69
Wealth status consistent	3.03	−1.90	7.97	0.31	−3.48	4.10	0.19	−3.56	3.95
Partnership dynamics									
Never married WHSA-I and WHSA-II	7.35	−2.29	17.00	1.78	−5.53	9.09	2.22	−5.12	9.55
Married^a^ WHSA-I and WHSA-II									
Separated, divorced or widowed WHSA-I and WHSA-II	1.68	−10.91	14.26	−4.84	−14.45	4.78	−4.97	−14.54	4.60
Never married in WHSA-I, married in WHSA-II	5.36	−1.74	12.47	4.17	−1.31	9.64	4.30	−1.10	9.70
Sep, div or wid in WHSA-II, married in WHSA-II	0.89	−6.91	8.70	0.61	−5.37	6.59	0.49	−5.44	6.42
Never married/married in WHSA-I, sep, div or wid in WHSA-II	0.48	−8.47	9.44	3.10	−3.75	9.94	2.93	−3.88	9.74
Change in residence WHSA-I and WHSA-II									
Yes	−1.58	−6.52	3.35	−1.14	−4.91	2.62	−1.20	−4.95	2.55
No^a^									
Religion									
Christian^a^									
Muslim	5.90	−3.78	15.57	−4.34	−11.77	3.10	−4.29	−11.65	3.07
Other	−2.08	−12.68	8.52	1.56	−6.51	9.63	1.81	−6.25	9.87
Highest Educational Attainment WHSA-I									
None	−7.74**	−15.11	−0.38	−2.57	−8.23	3.10	−2.91	−8.51	2.69
Primary	1.79	−4.82	8.39	3.13	−2.01	8.26	2.78	−2.24	7.81
JSS^a^									
SSS+	−1.48	−6.81	3.84	−1.78	−5.84	2.28	−1.77	−5.82	2.28
Ethnicity									
Ga^a^									
Akan	2.07	−3.96	8.10	3.10	−1.59	7.80	2.65	−1.93	7.23
Ewe	3.11	−4.58	10.80	3.26	−2.58	9.10	2.57	−3.27	8.42
Other	−4.91	−14.98	5.16	0.12	−7.64	7.87	−0.14	−7.80	7.52
Born in the Greater Accra region									
Yes	4.79*	−0.37	9.94	4.91**	0.99	8.84	4.97**	1.05	8.89
No^a^									
Age at WHSA-I	0.06	−0.44	0.56	0.23	−0.15	0.61	0.27	−0.11	0.65
Relationship to household head									
Head	−5.23	−2.69	13.14	−6.06*	−12.20	0.09	−6.09**	−12.11	−0.08
Wife^a^									
Daughter/daughter in-law	−12.92***	−21.71	−4.12	−8.62**	−15.33	−1.91	−8.69**	−15.38	−2.01
Other	−7.77	−17.19	1.65	−11.32***	−18.72	−3.92	−11.37***	−18.53	−4.21
Not specified	−11.78**	−22.48	−1.08	−3.24	−11.39	4.91	−3.01	−11.15	5.13
Household demographics									
Number of children aged 0–5 years									
0^a^									
1	2.89	−2.21	7.99	0.94	−2.92	4.81	0.73	−3.15	4.61
2+	−1.17	−8.24	5.90	−4.74*	−10.13	0.64	−4.91*	−10.28	0.47
Number of children aged 6–11 years									
0^a^									
1	0.16	−5.30	5.63	3.23	−0.94	7.40	3.19	−0.97	7.34
2+	−6.67*	−13.75	0.41	1.96	−3.52	7.43	2.48	−2.90	
Number of females aged 12–15 years									
0^a^									
1+	−0.36	−6.40	5.68	−1.76	−6.34	2.82	−2.01	−6.60	2.59
Number of males aged 12–15 years									
0^a^									
1+	−1.52	−8.44	5.39	−4.01	−9.23	1.20	−4.39	−9.64	0.87
Number of females aged 16–54 years									
1^a^									
2	5.17	−1.74	12.08	4.68*	−0.60	9.96	5.00*	−0.26	10.25
3	9.08**	1.42	16.73	5.09**	−0.81	10.99	5.46*	−0.36	11.28
4+	1.45	−6.28	9.18	4.15	−2.23	10.53	4.23	−1.64	10.11
Number of males aged 16–54 years									
0	2.28	−3.26	7.81	5.02**	0.78	9.26	5.01***	0.80	9.22
1^a^									
2+	9.39**	1.73	17.05	7.51**	1.60	13.43	7.70***	1.87	13.52
Number of females aged 55 years+									
0^a^									
1+	3.26	−2.78	9.31	5.07**	0.43	9.71	4.87**	0.27	9.46
Number of males aged 55 years+									
0^a^									
1+	2.69	−3.88	9.27	−0.69	−5.69	4.32	−0.42	−5.42	4.58
Constant	−9.96	−29.52	9.60	−12.84	−28.02	2.35	−15.45	−30.32	−0.58
IMR	−0.64	−11.96	10.69	1.86	−7.55	11.27	4.57	−4.04	13.18

*JSS* Junior Secondary Schooling, *SSS* Senior Secondary Schooling, *SM1* inverse Mills ratio calculated using selection model 1, *SM2* inverse Mills ratio calculated using selection model 2, *SM3* inverse Mills ratio calculated using selection model 3

*** Significance at the 1 % level, ** 5 % level, * 10 % level

^a^Reference category

When considering change in mental health, education, relationship to the household head and the number of males and females aged 16–54 years present in the household at WHSA-I were statistically significant at the 5 % level (Table 4). Women with no education experienced a less positive change in their mental health compared to women with JSS education (though the mean change in scores did not differ significantly between women with JSS education and those with primary or SSS or higher education). Compared with women who were the wives of household heads those who were daughters (or daughters in-law) or those who had a relationship not specified experienced a less positive change in their mental health. Women who, at WHSA-I, were resident in a household containing three women aged 16–54 years experienced a more positive change in their mental health compared to women who were resident in a household with only one woman of that age group. Lastly, women who were present in a household where two or more men aged 16–54 years were present experienced a significantly more positive change in their mental health compared to women in households with only one man of this age. It should be noted these relationships were not significant when considering working women without a child born in the survey interval.

When considering change in physical health, women who were born in the Greater Accra region experienced a more positive change in their physical health than those born elsewhere. As with mental health, in comparison to women who were the wives of household heads those who were daughters (or daughter in-laws) or those who had relations defined as other experienced a less positive change in their physical health. Women who at WHSA-I were resident in households containing three women aged 16–54 years experienced a more positive change in their physical health compared to women who were resident in a household with only one woman in this age group. Women who were present in a household where two or men aged 16–54 years were present experienced a significantly more positive change in their physical health than women in households with only one man of this age. However, in the models with SM2 and SM3, women living in households with no males aged 16–54 years present also experienced a more positive change in their physical health compared to where one man of this age was present. When looking at the models with SM2 and SM3, the presence of women aged 55 years and older in the household is also associated with a more positive change in physical health. It should be noted these relationships were not significant when considering working women without a child born in the survey interval.

## Discussion

### Impact of Combining Economic Work and Childbearing

Despite concerns about the negative consequences of maternal multiple roles in Sub-Saharan Africa, this study indicates there are no health penalties of combining work with having young children among women in Accra, Ghana. In our sample of working women change in both physical and mental health between WHSA-I and WHSA-II did not differ significantly between women who had a child alive at the second wave born in the survey interval, and women who did not. This result was found regardless of the selection specification used to control for attrition between the two waves, included in the model. Ahmed-Nia’s [3] study in Tehran, which compared non-working and working mothers, attributed a non-significant finding partly to the weight placed on women’s roles as wives and mothers in Muslim culture. In Ghana, despite past fertility decline, biological motherhood is still seen as the primary role in a woman’s life and is valued socially [46]. Through contributing to women’s gendered identity and social interaction, childbearing could promote health and wellbeing, this effect counteracting the strain of combining work with young children.

We also found that having to combine work and young children did not influence health through causing a change in wealth. In the Ghanaian context the meaning of motherhood is being able to provide for children, and consequently the engagement of women in economic activity is an important criterion in fulfilling the responsibility of being a mother [12]. This economic role of biological mothers could explain the finding of the birth incurring no shocks to the household economy. Additionally, strain might be reduced due to working mothers being perceived as good mothers. Activities of mothering are also not the sole responsibilities of biological mothers with female kin, especially grandmothers, being identified by the literature as important sources of care [14].

Another explanation for working mothers’ health balance could include the adjustment of individuals’ expectations and definitions of health as a result of their changing circumstances (becoming a mother to a young child). Qualitative fieldwork undertaken by the first author found that, while mothers of young children in Accra reported a decline in their health relative to before having their youngest child, they emphasized that this was the norm for all mothers. Mothers of young children may change how they rate their health seeing implications for their wellbeing as a transitional reality of their role.

### The Mediation of Socio-economic and Household Demography and Maternal and Economic work Performance Among Women with Young Children

Relationship to the household head was found to have the largest influence on change in both mental and physical health among women who gave birth in the survey interval. Relationship to the household head is a marker of an individual’s status in the household [17]. Status influences bargaining position, affecting the tasks assigned to individuals and the resources they receive. A difference in bargaining power or roles, and consequent inequality in the distribution of tasks and resources between wives compared to daughters (in-law) and those of a relation defined as other or as a relation not specified may be responsible for the less favourable change in health among the latter groups.

The number of males and females in the household at the WHSA-I was also associated with both change in mental and physical health. The number of males and females aged 16–54 years in the household could be indicators of social support. Co-residence with a greater number of adults may provide greater opportunities for the sharing of productive and domestic responsibilities [27]. Where women have new maternal demands, the presence of other adults in the household may allow for the reallocation of tasks to ensure the health of mothers and children. Similarly, women born in the Greater Accra region may have greater family ties in the area and consequently sources of emotional and instrumental support to call upon.

Education was only found to be associated with change in mental health among women who had a child born in the survey interval. In Accra maternal education has been found to be a vital determinant of good care among women with young children in terms of feeding, use of preventative health services and hygiene practices [7]. Knowledge of appropriate childcare methods may influence the mental health of mothers directly through increasing their self-esteem and confidence in their mothering abilities, and indirectly through its influence on child development.

### Limitations

Whilst the longitudinal nature of this study could be considered a strength, the reliance on two waves of data was a limitation in that change in parent status and change in health were explored in the same time period. To have had three or more waves of data would have allowed a time lag between the measurement of our covariate of interest and our outcome [18]. Furthermore, whilst the analysis was restricted to women working at both time points it was unknown whether women were working consistently through the survey interval. It is likely that temporary withdrawal from economic activity is a common strategy of mothers after childbirth. The timing of the resumption of economic activity may have important consequences for maternal health that are not examined by this study. In relation to women’s work, an additional important missing dimension, due to the small sample size, was the nature of female work, such as working hours and sector or type of employment, which may mediate the relationship between women’s role performance and health. Lastly, this analysis might be sensitive to the dimension and measurements of health considered.
